# Malaysian and Chinese King Cobra Venom Cytotoxicity in Melanoma and Neonatal Foreskin Fibroblasts Is Mediated by Age and Geography

**DOI:** 10.3390/toxins15090549

**Published:** 2023-09-04

**Authors:** Bianca Op den Brouw, Manuel A. Fernandez-Rojo, Tom Charlton, Bryan G. Fry, Maria P. Ikonomopoulou

**Affiliations:** 1 Venom Evolution Lab, School of Biological Sciences, The University of Queensland, Brisbane, QLD 4072, Australia; b.m.opdenbrouw@gmail.com; 2 Hepatic Regenerative Medicine Group, Madrid Institute for Advanced Studies in Food, E28049 Madrid, Spain; mafernaro@hotmail.com; 3 Diamantina Institute, The University of Queensland, Brisbane, QLD 4072, Australia; 4 Department of Natural Sciences, Manchester Metropolitan University, All Saints Building, Manchester M15 6BH, UK; tom@ecoanimalencounters.co.uk; 5 Translational Venomics Group, Madrid Institute for Advanced Studies in Food, E28049 Madrid, Spain; 6 Institute for Molecular Bioscience, The University of Queensland, Brisbane, QLD 4072, Australia

**Keywords:** cobra snakes, animal venom, cytotoxicity

## Abstract

Snake venoms constitute a complex, rapidly evolving trait, whose composition varies between and within populations depending on geographical location, age and preys (diets). These factors have determined the adaptive evolution for predatory success and link venom heterogeneity with prey specificity. Moreover, understanding the evolutionary drivers of animal venoms has streamlined the biodiscovery of venom-derived compounds as drug candidates in biomedicine and biotechnology. The king cobra (*Ophiophagus hannah*; Cantor, 1836) is distributed in diverse habitats, forming independent populations, which confer differing scale markings, including between hatchlings and adults. Furthermore, king cobra venoms possess unique cytotoxic properties that are used as a defensive trait, but their toxins may also have utility as promising anticancer-agent candidates. However, the impact of geographical distribution and age on these potential venom applications has been typically neglected. In this study, we hypothesised that ontogenetic venom variation accompanies the morphological distinction between hatchlings and adults. We used non-transformed neonatal foreskin (NFF) fibroblasts to examine and compare the variability of venom cytotoxicity between adult captive breeding pairs from Malaysian and Chinese lineages, along with that of their progeny upon hatching. In parallel, we assessed the anticancer potential of these venoms in human-melanoma-patient-derived cells (MM96L). We found that in a geographical distribution and gender-independent manner, venoms from hatchlings were significantly less cytotoxic than those from adults (NFF; ~Log EC_50_: 0.5–0.6 vs. 0.2–0.35 mg/mL). This is consistent with neonates occupying a semifossorial habitat, while adults inhabit more above-ground habitats and are therefore more conspicuous to potential predators. We also observed that Malaysian venoms exhibited a slightly higher cytotoxicity than those from the Chinese cobra cohorts (NFF; Log EC_50_: 0.1–0.3 vs. 0.3–0.4 mg/mL), which is consistent with Malaysian king cobras being more strongly aposematically marked. These variations are therefore suggestive of differential anti-predator strategies associated with the occupation of distinct niches. However, all cobra venoms were similarly cytotoxic in both melanoma cells and fibroblasts, limiting their potential medical applications in their native forms.

## 1. Introduction

Venom is generally defined as a functional trait: a complex secretion produced by a specialised gland tissue that is delivered into the body of another animal via a wound for use in antagonistic interactions [1]. By this biological definition, the majority of snake species are venomous. However, while the concept of “a venomous snake” usually invokes medical notions of significant danger to humans, the toxic secretions of most snakes are mild, and only a small proportion poses a serious threat to humans. Furthermore, the innumerable bioactive molecules (i.e., toxins) comprising these secretions possess pharmacological properties, such as analgesic, metabolic, immunomodulating, anti-thrombotic or anticancer ones, that can be exploited as therapeutics and developed into life-saving medicines [2,3].

The scale of mining venoms for drug candidates is immense, as this mixture often contains hundreds of toxins and isoforms that undergo accelerated rates of evolution [4]. This biochemical complexity underpins the striking diversity in toxin expression found across species, populations, and even within individuals throughout their lifetime [5]. This variability deepens the reservoir of pharmacological potential while highlighting new medical applications [6].

Studying the natural history and evolution of venomous species provides key insights regarding the biological sources reflecting toxin diversity. Snake venoms function at the ecological and biochemical interface between predator and prey, aiming mainly to facilitate predation [7]. This has led investigations of drivers in snake venom variation to focus on prey preferences [6,8,9,10]. However, this approach overlooks the ecological complexity that underlies the evolution of a phenotype. Numerous biotic and abiotic factors, such as behaviour, morphology, prey diversity, population size, biogeography, and climate, variably interact to produce a “fingerprint” of macro- and microevolutionary processes within a population. Consequently, it shapes the biochemical architecture of both predator and prey. Accordingly, inter-populational differences in any of these components can be indicators of cryptic diversity [11,12,13,14,15,16].

**Figure 1:**
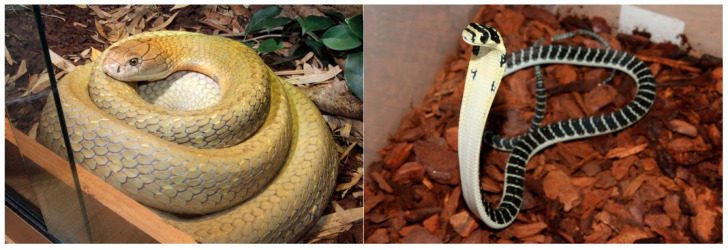
Representative image of adult (**left**) and juvenile (**right**) Malaysian king cobra provided by Tom Charlton.

The king cobra (*Ophiophagus hannah*; Cantor, 1836) belongs to the Elapidae family and is widely distributed from India to southern China and Southeast Asia in numerous geographically isolated mainland and island populations. These populations form at least four genetically distinct lineages, which are likely separate species [17]. They occupy a diversity of ecotypes across their distribution, including rainforest, alpine forest, plantations, paddy fields, and mangrove swamps, across tropical, subtropical, and temperate climes [18,19,20]. Morphological variation in coloration and banding is evident between lineages, as well as between juveniles and adults (Figure 1) and between sexes for some localities [21].

The king cobra is not a “true” cobra (*Naja* sp.), despite being named as such. This misnomer likely derives from the cobra-like hooding behaviour that it adopts when feeling threatened, its immense length of 5–6 metres [18], and its tendency to feed primarily on other snakes—including highly venomous species [18]. Adults yield tremendous quantities of venom dominated by neurotoxic three-finger-toxins (3FTx), along with lesser quantities of toxins such as phospholipases (PLA_2_), snake venom metalloproteases (SVMP), and L-amino acid oxidases (LAAO), the latter of which are evolutionarily distant from those of other snake LAAOs and have shown promise as anticancer agents [22,23,24,25,26]. Venom variation between individuals within Thailand has been documented, as well as between pooled venoms of adults from Thai, Malaysian, Chinese, and Indonesian populations, with significant variation in antivenom effectiveness [27,28,29]. Notably, the Malaysian population has been shown to be the most cytotoxic [12] and least neurotoxic [30]. Distinctions between the venoms of juveniles and adults or between genders are still lacking for any geographical population.

The king cobra complex represents an ecologically and toxicologically interesting group that harbours a significant degree of venom diversity and pharmacological potential. We hypothesised that ontogenetic venom variation accompanies morphological alterations evident between adult and hatchling snakes as a reflection of differences in niche occupation. In this study, we examined the cytotoxicity of juvenile and adult king cobras from Chinese and Malaysian lineages using neonatal foreskin (NFF) cells and discussed these results in the context of king cobra ecology. We additionally assessed their anticancer potential in human melanoma (MM96L) cells.

## 2. Results

**Figure 2:**
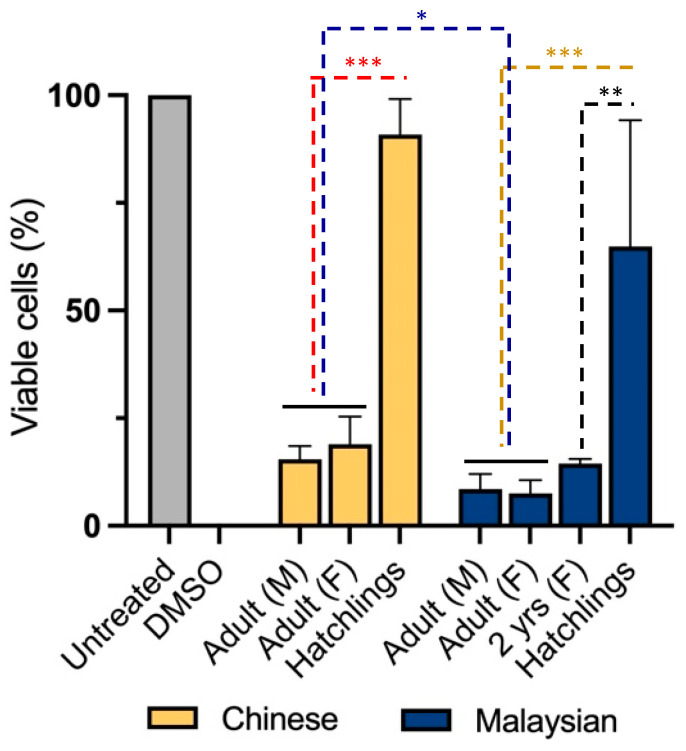
Remaining proportion (%) of viable neonatal foreskin (NFF) cells after 24 h of incubation with 2.5 µg/mL of king cobra (*Ophiophagus hannah* sp.) venoms from Malaysian (dark blue) and Chinese (yellow) lineages as well as untreated cells (grey)quantified using MTT assays. Data are presented as mean ± SD and are the result of at least three replicates, * *p*  <  0.05, ** *p* < 0.0002 & *** *p*  <  0.0001. Data are normalised to the vehicle (negative) control (100% viability); 10% DMSO was used as a positive control.

To examine cytotoxicity in non-transformed cells, we treated neonatal foreskin fibroblasts (NFFs) with venoms for 24 h at 2.5 µg/mL. It was shown that gender was not a significant factor determining the diversity of cytotoxicity between Chinese and Malaysian king cobras (Figure 2). Indeed, venoms from adult male and female snakes demonstrated high levels of cytotoxicity, with the Chinese venoms reducing proportions of viable cells down to 15 ± 3.0% (male) and 19 ± 6.4% (female) (unpaired *t*-test: t(4) = 0.8599, *p* = 0.438353) and those of Malaysia doing so down to 9 ± 3.5% (male) and 8 ± 3.1% (unpaired *t*-test: t(4) = 0.3689, *p* = 0.730875) (Figure 2). 

However, venom-induced cytotoxicity was mediated by age and geographical distribution in cobras. Unlike adults—females and males—venom from hatchlings exhibited less impact on NFF cell viability (Chinese King Cobra, 91 ± 8.2%; Malaysian King cobra, 65 ± 29.3% of viability (two-way ANOVA: F_3,16_ = 40, *p* < 0.0001)). This difference was already apparent when we assessed the venom of a juvenile (2 year old) Malaysian snake. The latter reduced cell viability to 14 ± 1.0%, a similar cytotoxicity to that observed in adult snake venoms (Tukey’s, *p* = 0.0002). Moreover, geographical origin was found to be a significant co-factor in cytotoxicity but a weaker source of venom diversity. Indeed, Malaysian venoms exhibited a more profound cytotoxicity than their Chinese counterparts (two-way ANOVA: F_1,16_ = 6.892, *p* = 0.0184).

**Figure 3:**
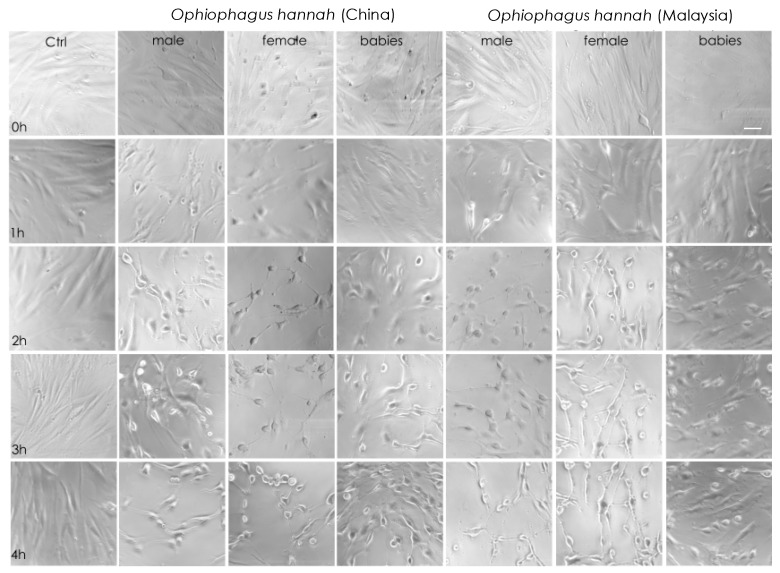
Light-microscopy representative images of neonatal foreskin fibroblast (NFF) cells at 63× magnification, incubated at 0–4 h with 50 µg/mL of king cobra (*Ophiophagus hannah* sp.) venoms from Chinese and Malaysian lineages. Scale bar = 20 µm.

Cell imaging of NFF cells with light microscopy during the first four hours of incubation with cobra venoms showed that at high concentrations (50 μg/mL), their cytotoxicity was obvious. Indeed, there was evidence of cell stress, such as changes in cellular morphology that exhibited a rounder shape and lower confluency, which translated to decreased cell-to-cell contact and lesser cell adhesion after one hour of incubation. These symptoms were more apparent after two hours in adult venoms from both species (Figure 3). Interestingly, although cell imaging showed that venoms from hatchlings exhibited cytotoxicity at high concentrations, cells were visually less affected at 4 h of treatment in comparison to adult snake venoms. A limited effect in the viability of non-transformed NFF cells is an essential asset of anticancer drug candidates. In our study, this was evident in hatchlings’ venoms. Therefore, venom samples were assessed further by increasing the incubation period to 48 h in both healthy (NFF) and melanoma (MM96L) cells at a range of venom concentrations.

**Figure 4:**
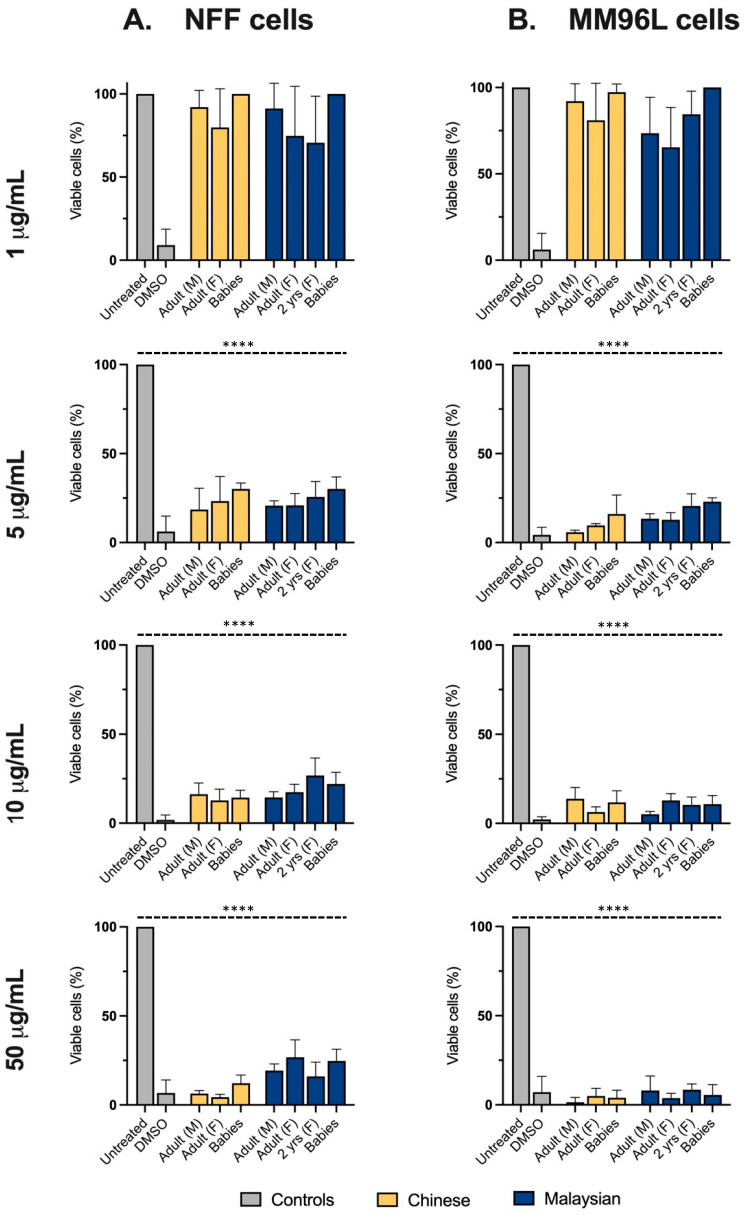
The proportion (%) of viable neonatal foreskin fibroblasts (NFFs) (Column (**A**)) and melanoma (MM96L) cells (Column (**B**)) after 48 h of incubation with 1–50 μg/mL of king cobra (*Ophiophagus hannah* sp.) venoms from Malaysian (dark blue) and Chinese (yellow) lineages, quantified using MTT assays. Data are presented as mean ± SD and are the result of at least three independent experiments, performed in triplicates and normalised to vehicle controls (100% viability). All treatments were compared to untreated (control, grey) cells. **** *p*  <  0.0001.

At concentrations of ≥5 μg/mL, all venoms, including those from hatchlings, diminished the viability of melanoma cells to fewer than 25%, showcasing their potency (Figure 4B). Of interest, at this incubation period, the age-associated variations previously observed at 24 h of incubation were greatly reduced or negated in non-transformed fibroblasts. Indeed, all venoms equally decreased the proportion of viable NFF cells to ≤30% at 48 h (Figure 4A), but their cytotoxicity tended to be higher in melanoma cells, indicating a preference for targeting tumorigenic cells. 

At high venom concentrations and at an increased incubation period, the geographic-associated variations in cytotoxicity in NFF cells observed at 2.5 μg/mL were also greatly lessened. However, the EC_50_s calculated from the dose-response data indicated that the Malaysian venoms were, broadly speaking, slightly more potent than their Chinese counterparts in both cell lines (Table 1). Although venoms generally diminished the viability in melanoma cells to a slightly greater extent than in fibroblasts, their EC_50_s implied a similarly high potency. 

## 3. Discussion

Venoms from the Malaysian king cobras generally demonstrated a greater cytotoxicity than their Chinese counterpart snakes, which is consistent both with the Malaysian population being more strongly marked with a defensive colouration and with previous studies showing a higher level of cytotoxicity and consequent lower levels of neurotoxins present in the venoms [12,30]. Of interest, the venom of hatchlings from both localities possessed significantly weaker cytotoxic properties than that of adults. This is in accordance with their relative conspicuousness to predators, with juveniles being semi-fossorial, while adults are more active in the open and therefore more likely to encounter potential predators. In addition, most venoms demonstrated a slightly greater, but not significant, potency in melanoma when compared with non-transformed fibroblasts. As crude venoms were used, the observed cell cytotoxicity could be the result of a potent toxin or the synergistic effect of two or multiple toxins. However, the observed broad-spectrum potency in healthy and cancerous cells suggests that any anticancer therapeutic potential of these venoms should be formulated with direct delivery approaches into tumors. Indeed, a limitation of investigating the venom lies in the quantity needed to extensively characterise it. In addition, the complexity of the crude venom’s components prompts one to identify the toxin reflecting the biological activity. However, an even more promising strategy is the chemical synthesis of the toxin of interest. Chemically synthesised compounds could be further optimised for their efficacy and safety and could therefore set the foundations for the next generation of anticancer drug candidates. Promising examples of such toxins targeting various tumor cells are reported in the literature, including peptides inspired from Lys49 phospholipase A2 snake venoms [31,32,33]. 

While king cobra venoms do possess cytotoxic 3FTxs (CTX), LAAOs are primarily responsible for their cytotoxicity [34,35]. King cobra LAAOs are exceptionally potent and are known to possess a number of unique drug assets, including heat stability and retention of activity over extended periods of time [24,36]. Accordingly, the potency of LAAOs in hatchlings’ venoms at 48 h could account for the observed cell death. 

Age is a common factor of venom variation among snakes and is often linked to parallel shifts in prey and, potentially, diet [37]. Unfortunately, little knowledge exists on the ecology of hatchling king cobras, including their diet composition. The literature suggests that cytotoxins in hooding Asian and African elapids are defensive, rather than predatory, venom components [11,12]. Previously, we reported that cytotoxicity evolved primarily as a defensive innovation and that it has co-evolved twice alongside hooding behavior and independently of spitting [12]. Newly hatched king cobras are markedly more distinct in patterning and coloration than adults and are thought to be largely arboreal during the first year of their life, transitioning to a semi-arboreal lifestyle between 1–2 years of age, and becoming mostly terrestrial as adults, though they may also hunt arboreally [38]. The temporal increase in venom cytotoxicity observed in this study generally coincides with these shifts in niche occupation. With the exception of instances of cannibalism, large adult king cobras have few natural predators, though subadults may be vulnerable to predation by terrestrial carnivores [39], with species such as mongooses (Herpestidae) and hog badgers (Mustelidae) showcasing exceptional skills in overpowering venomous snakes and resistance to snake venom neurotoxins [40]. Consequently, pain-inducing cytotoxins form a crucial line of defence against such mammalian predators, in this instance during the transitional period from the occupation of arboreal to terrestrial environments. Juveniles may be easily overcome by predators of sufficient size, including carnivorous birds, monitor lizards, and even other snakes [38]. Cytotoxic venom may therefore be of limited defensive use to hatchlings, who likely rely instead on predator avoidance through their arboreality and the crypsis conferred by their distinct banding in these light-mottled arboreal habitats. 

The extent of dietary differences among king cobra populations is understudied, though divergence in prey preference (e.g., from primarily snakes to primarily monitor lizards) has been observed in some localities [41], along with significant differences in taxon-specific venom lethality among populations [29]. Further studies into ontogenetic variations in the ecology and venom difference of these snakes are needed to test this hypothesis, though assessing ontogenetic and geographic differences in neurotoxicity alongside cytotoxicity may offer some broad insights into the role of predatory versus defensive venom components. 

While gender was not found to be a factor of venom variation in this study, no firm conclusions can be drawn on this variable, as only one adult of each gender was tested for each location, and venom is known to vary significantly among individuals [29]. Such individual variation is perhaps unsurprising, given the discrepancies between habitat preference and home range found between individuals of a given locality [20,41]. However, sexual dimorphism and sex-linked differences in behaviour are evident within king cobras, and therefore broad gender-linked trends in venom composition may also occur. Increasing the sample size is necessary to adequately test this hypothesis. 

The king cobra complex contains venom variation that is worthy of concerted research attention. Although the complex is yet to be taxonomically revised, the venom variation observed in this study reinforces the need to clearly define origin and age in snake samples in future work. Research on the ecology and venom composition of each lineage of these snakes is essential for a further assessment of their pharmacological potential and for the conservation of these biologically important snakes.

## 4. Conclusions

We observed that age was a contributor of cytotoxicity in Malaysian and Chinese king cobra. We also reported that the venom of hatchlings was significantly less potent than that of adults, based on the cytotoxicity results in fibroblasts. This is in alignment with their ecology, where neonates are born as arboreal before transitioning to a semi-arboreal lifestyle and finally transforming into terrestrial adults. The temporal increase in venom cytotoxicity coincides with these shifts in niche occupation, which reflect their defensive behaviour as adults. Therefore, this work sheds light on the association between ecology and cytotoxicity in king cobras. It also generates new knowledge on the impact of geographical distribution, probably due to diet preferences, on venom cytotoxicity. From a therapeutic discovery perspective, the observation that venom from hatchlings is less cytotoxic than that from adults in fibroblasts, but still effective at targeting melanoma cells, might suggest that the former could be more suitable for further evaluation and development as a drug candidate. 

## 5. Materials and Methods

### 5.1. Venoms

Venoms were obtained from captive specimens of *O. hannah* from a private collection in the UK. All venom study protocols in this work were performed with the University of Queensland Biosafety Approval #IBC134BSBS2015 and University of Queensland Animal Ethics Approval 2021/AE000075. Pairs of adult snakes from Malaysian and Chinese lineages were bred in captivity. The venom from the adults and that of their young was extracted within approximately eight weeks after hatchlings emerged. Additionally, venom was extracted from a 2 year old captive-bred Malaysian female king cobra hatched from a previous breeding by the same adult pair of Malaysian adults. Venoms were immediately flash-frozen in liquid nitrogen, lyophilised, and stored at −80 °C. Prior to testing, the venoms of sibling hatchlings were reconstituted in deionised water and pooled. 

### 5.2. Cytotoxicity Analyses

We used human melanoma cells (MM96L) and human neonatal foreskin fibroblasts (NFFs), supplied by QIMR Berghofer Medical Research Institute. Cells were maintained in RPMI medium supplemented with 1% penicillin–streptomycin and foetal calf serum (FCS), as previously described [6,42,43]. Cell viability was evaluated using colourimetric MTT (Thiazolyl Blue Tetrazolium Bromide; Sigma-Aldrich, St Louis, MO, USA) assays [6,42,43]. The following venom concentrations of 1 µg/mL, 2.5 µg/mL, 5 µg/mL, 10 µg/mL, or 50 µg/mL were added to cells (*n* = 4) and incubated for 24 h or 48 h. We used 10% of dimethyl sulfoxide (DMSO) as a positive control (100% toxicity). The absorbance was read at 570 nm. We performed three independent experiments with a minimum of three replicates per treatment. The readings from all treatments were normalised against their untreated control cells, which were considered as 100% viability and then subtracted from wells only containing media.

### 5.3. Light Microscopy

Cells were plated in 24-well plates (8000/well) and cultured for 0 to 4 h in the presence or absence of Chinese or Malaysian (babies and adults of both genders) cobra venoms at a concentration of 50 µg/mL. We conducted three independent experiments of three replicates each. Then, images of the cells were taken at the mentioned times using a Leica DMIL LED epifluorescence microscope and using the LAS V413 software version number LASV4.13.0. Panel figures were set using ADOBE photoshop CS3.

### 5.4. Statistics

Data were graphed and analysed using GraphPad Prism version 9 for iOS (GraphPad Software, San Diego, CA, USA). For the MTT assays, venom cytotoxicity data were normalised to the vehicle-treated cells (100% cell viability) to calculate the percentage of viable cells following venom treatment. Data were then tested for normality using Shapiro–Wilk tests (significance set to *p* < 0.05). To assess differences between genders, the percentages of viable cells were compared using unpaired *t*-tests (males vs. females), since data were normally distributed. As no difference was evident, cell viability data from adult snakes were combined for subsequent statistical analyses. Two-way ANOVAs with Tukey’s post hoc comparisons were then run to evaluate geography and age-linked venom variation. To calculate venom EC50s, dose-response data were transformed (venom concentrations log-transformed) and normalised (smallest mean 0%, largest mean 100%), and then curves were subjected to non-linear regression using the [log(agonist) vs. normalised response − variable slope] prism function. 

## Figures and Tables

**Table 1:** Venom cytotoxicity (EC50_s_) calculated from the remaining proportion (%) of viable neonatal foreskin fibroblasts (NFFs) and melanoma (MM96L) cells at 48 h of incubation with 1–50 μg/mL of king cobra (*Ophiophagus hannah* sp.) venoms derived from Malaysian and Chinese lineages and quantified using MTT assays. Data are the result of best-fit values, following non-linear regression of at least three independent experiments conducted in three replicates.

	Log EC_50_ (μg/mL)			
	Chinese		Malaysian	
	MM96L	NFF	MM96L	NFF
Adult (M)	0.3 ± 0.06	0.4 ± 0.05	0.2 ± 0.07	0.3 ± 0.07
Adult (F)	0.2 ± 0.08	0.3 ± 0.08	0.2 ± 0.07	0.1 ± 0.09
2 years (F)	N/A	N/A	0.3 ± 0.05	0.2 ± 0.10
Hatchlings	0.3 ± 0.08	0.5 ± 0.04	0.5 ± 0.05	0.6 ± 4.60 *

* ambiguous fit.

## Data Availability

The data presented in this study are available upon request to the corresponding authors.
